# Examination of the Knowledge Levels of Family Medicine Residents in Turkey about Oral and Dental Health

**DOI:** 10.3290/j.ohpd.b5656296

**Published:** 2024-08-06

**Authors:** Sümeyye Tuncer Hancı, Eda Güler, Önder Hancı

**Affiliations:** a Restorative Dentistry Specialist, Restorative Dentistry Department, Bafra Oral and Dental Health Hospital, Samsun, Turkey. Wrote the manuscript, prepared and distributed the questionnaire.; b Professor and Medical Doctor, Restorative Dentistry Department, Ondokuz Mayıs University, Faculty of Dentistry, Samsun, Turkey. Study design.; c Family Medicine Specialist, Family Medicine Department, 19 Mayıs County State Hospital, Samsun, Turkey. Evaluated the questionnaire, performed statistical analysis.

**Keywords:** community dental health, family medicine, oral and dental health, preventive dentistry, preventive medicine

## Abstract

**Purpose:**

To measure the general oral and dental health knowledge level of family medicine residents who are receiving full-time specialty training in Turkey. Primary care physicians can contribute to improving the oral and dental health of patients during general health services.

**Materials and Methods:**

The fundamentals of oral and dental health that the family medicine physicians should know about were determined, and questionnaire items on these fundamentals were prepared. The sample size was calculated as 296 individuals. The survey was conducted online. The collected data were analysed employing the following tests: chi-squared, Fisher, Kolmogorov-Smirnov, Spearman, ANOVA, Mann-Whitney U, Kruskal-Wallis, and Bonferroni.

**Results:**

302 family medicine residents in various clinics in Turkey participated in the study. The mean age of the participants was 29.6 ± 5.1. The mean knowledge scores of the resident physicians were calculated as 65.2 ± 10.9 (lowest: 27; highest: 92). The majority of resident physicians stated that they did not receive training on oral and dental health during their residency training, and that they agreed with the idea of integrating it into the residency training curriculum.

**Conclusions:**

The general knowledge level of family medicine residents in Turkey about oral and dental health was found to be moderate.

Family medicine is in a better position than dentistry in terms of reaching more patients, as it is the gateway for patients to the health system. In Turkey, each individual has a family physician. For this reason, it is very important provide preventive oral and dental health services, especially to those who are not aware of the importance of oral and dental health and who do not regularly attend dental appointments, as well as to disadvantaged patient groups who have difficulty accessing a dentist.^7,22^

Oral and dental health should not be ignored during periodic examinations by the family physician. However, in practice, oral and dental health is not yet sufficiently integrated into family medicine in Turkey. Similarly, oral and dental health is unfortunately not included in the curriculum of family medicine residency programs. Preventive oral and dental health services are carried out by Community Health Centers, Oral and Dental Health Centers and Faculties of Dentistry in Turkey. However, it is not possible for them offer service coverage as widely as the Family Health Centers in every district. It is recommended to train those health service providers who already have the opportunity to deliver preventive services instead of hiring new staff, which would increase costs. Reform which integrates preventive oral/dental health care into the family medicine system is necessary to reach the target population.

As part of a holistic approach to the patient, family medicine specialists should be able to monitor the oral and dental health of the patients, give the necessary recommendations and direct them to a dentist when necessary. This study aimed to determine the level of knowledge of the resident physicians enrolled in the residency program in the branch of family medicine in Turkey on oral and dental health and their views on the curriculum of family medicine residency programs and practice.

The null hypothesis of the study is that the level of knowledge about oral and dental health of family medicine residents in Turkey is moderate.

## MATERIALS AND METHODS

Written permission (date: 25/06/2020; decision number: 2020/412) was obtained from the Ondokuz Mayıs University Clinical Research Ethics Committee for the study. For this cross-sectional survey study, data were collected from family medicine clinics of medical faculties and training and research hospitals in Turkey via an online questionnaire. Before participating in the study, resident physicians were informed about the study online.

By examining previous studies publications on family medicine and oral and dental health, the fundamentals that the family medicine physicians should know about oral and dental health were determined, and the questionnaire was correspondingly prepared.^4,19,25,27,30^ With this questionnaire (see supplementary material), the knowledge levels and sociodemographic characteristics of the resident physicians about oral hygiene, systemic diseases, mother-child-pregnancy and oral-dental health were determined. According to the sample calculation made, the minimum number of resident physicians required to participate in the study was n=296. The questionnaire was distributed to the participants through the social network of resident representatives from all institutions providing family medicine education in Turkey. Participants from a total of 30 institutions from all regions of the country answered the online questionnaire. There were at least 10 participants from each institution. The number of resident physicians participating in the study was 302.

The data obtained from the study were entered into SPSS (Statistical Package for Social Sciences) for Windows 25.0 (SPSS Inc, Chicago, IL) and statistical analysis was performed. Descriptive statistics were presented as mean ± SD, min/max values, frequency distribution and percentage. Pearson’s chi-squared test, a continuity-corrected chi-squared test and Fisher’s exact probability test were used to evaluate categorical variables. The normality of the quantitative variables was tested using a histogram, coefficient of variation, skewness and kurtosis level, and the Kolmogorov-Smirnov Test. Spearman’s correlation test was used as a statistical method to determine statistical significance between two quantitative variables. For the variables found to fit the normal distribution, and for statistical significance between two independent groups, the t-test was used in independent groups and one-way ANOVA was used in groups with more than two independent variables. For the non-normally distributed variables, the Mann-Whitney U-test was applied to determine statistical significance between two independent groups, and the Kruskal-Wallis test was used for groups with more than two independent variables. Bonferroni’s post-hoc test was also used. Statistical significance level was accepted as p<0.05.

In the questionnaire, a total of 41 questions were asked in 5 groups. The first group contained items on age, gender, and other sociodemographic characteristics. The second group asked questions about providing oral hygiene, such as brushing and flossing. The third group contained questions on the relationship between oral/dental health and systemic diseases affecting oral and dental health, such as diabetes and respiratory diseases. In the fourth group, under the title of mother-child-pregnancy, questions were asked about oral and dental health during pregnancy, and about ensuring and maintaining oral and dental health during infancy/childhood. The fifth group of questions addressed the attitudes and behaviours of the resident physicians on whether they received training in oral health during their undergraduate and specialist education, and about the integration of oral and dental health into primary health care institutions.

## RESULTS

302 individuals working as family medicine residents in various medical faculties and training-research hospitals in Turkey participated in the study. The mean age of the participants was 29.6 ± 5.1.

Questions about the frequency of dental check-ups and toothbrush changes were answered correctly at a low rate (7% and 27.2%, respectively). Oral hygiene questions were statistically significantly more often answered correctly by women (Table1).

**Table 1:** Correct answer scores for oral hygiene questions by gender

	Female (n=203)	Male (n=99)	p
Oral hygiene scores (mean±SD)	46.9±19.5	39.3±20.5	0.004*

The questions on the relationship between systemic diseases and oral and dental health were answered correctly at a high rate. The relationships between periodontal disease and premature and low birth weight, age at which fluoride toothpaste should be introduced, and transmission of bacteria from mother to baby, were the questions answered correctly at a low rate. There was no statistically significant difference between the sexes in terms of the answers given to the mother-child-pregnancy questions. Physicians who are married and have children gave correct answers to the mother-child-pregnancy questions statistically significantly more often (Table 2).

**Table 2:** Correlation of mother-child-pregnancy scores with marital status and having children

	Married (n=187)	Single (n=115)	p
Mother-child-pregnancy scores (mean±SD)	62.7±16.1	58.2±14.8	0.016*
	Has a child (n=90)	Has no child (n=212)	p
Mother-child-pregnancy scores (mean±SD)	68±14.8	58±15.3	0.000*


The answers to the questions aiming to measure the knowledge level of the resident physicians participating in the study were evaluated over 100 points. Physicians who marked the option “no idea” were considered to have given the wrong answer to the question. The mean knowledge score of the resident physicians was calculated as 65.2±10.9 (minimum: 27, the maximum: 92). The average scores calculated from the answers given by the physicians to the questions about oral hygiene, systemic diseases and mother-child-pregnancy are shown in Table 3.

**Table 3:** Average Scores of Resident Physicians

Question groups	Number of physicians (n)	Mean score (±SD)
Oral hygiene	302	44.5±20.2
Systemic diseases	302	90.3±14.4
Mother-child-pregnancy	302	61.1±15.8
Overall score	302	65.2±10.9

A “highly agree/strongly agree” response was received for the opinion that the family medicine specialist should also check the mouth and teeth during oropharyngeal examination. A “highly agree/strongly agree” response was found for the idea of providing oral dental care training by health provider in the family health center. A high percentage of “undecided” answers were received about the recommended fluoride application. For the opinion that oral and dental health screening and fluoride applications should be integrated into primary health care institutions, participants gave 41.1% “agree” and 27.5% “undecided” answers. The general scores of physicians who had previously worked in a family health center were found to be statistically significantly higher than those who did not (Table 4).

**Table 4:** Total scores according to status of work in family health center previously

	Worked (n=84)	Did not work (n=218)	p
Total scores (mean±SD)	68.1±10.8	64.1±10.6	0.004*

The resident physicians who recommended the use and/or application of fluoride to any patient who requested information about the use and application of fluoride were asked in which form(s) they recommend the use of fluoride. Fifty-three residents stated that they recommended the use of toothpaste, 11 residents recommended the use of varnish, 11 residents suggested the use of mouthwash, 2 residents recommended it to be added to drinking water, and 1 resident recommended it in tablet form.

The majority of the physicians could not receive adequate training in oral and dental health during their medical and family medicine training, but they wanted to receive it.

## DISCUSSION

Today, oral and dental diseases still affect a large part of the global population and constitute an important public health problem.^17^ At the same time, in addition to the general health and quality of life of individuals, it also negatively affects the economy with a serious increase in health expenditures.^1^

The aim of primary health care centers is to deliver preventive and curative health services to patients at the right time. The potential of family medicine to contribute to the protection of oral and dental health is quite high.^14,16^ For these reasons, it is critical that the general knowledge level of family medicine specialists about oral and dental health be such that these specialists can give preventive recommendations and direct patients to the dentist when necessary. For all these reasons, recent studies suggest the integration of oral and dental health into primary health care systems.^20,26,3^^2^

Previous publications have elucidated the basic information family medicine specialists should have about oral and dental health.^10,28,30^ Dental anatomy, infection (caries, pulpitis, abscess), periodontal diseases, dental traumas and preventive measures constitute the most important topics.^30^ The goal is to recognise the disease, decide when to refer, assess risk, provide oral health education and provide forward-looking guidance.^10^ In the study, whether this basic information is known or not and attitudes and thoughts about oral and dental health were examined.

Four questions aiming to measure residents’ knowledge level about oral hygiene were asked and the scores they obtained were calculated as a result of the answers they gave to these questions; their average was 44.5±20.2 points out of 100. In many other studies, similar to our study, the questionnaire method was used to determine the knowledge levels of family physicians and other medical doctors about oral and dental health.

In the study conducted by Oyetola et al^23^ on medical doctors, nurses and medical school students in Nigeria, the rate of correctly knowing the frequency of preventive dentistry visits was found to be only 6% among medical doctors.

Especially in countries like Turkey, where dental and gingival diseases are common in all age groups, preventive dentistry visits are of great importance for the prevention of many oral and dental diseases, especially caries and periodontal diseases; these visits should be at short intervals, such as 3-6 months.

Oral and dental health is an integral part of general bodily health. Systemic diseases affecting other tissues and organs in the body can negatively affect oral and dental health, and vice-versa, as primary diseases of the mouth and teeth can negatively affect the normal functions of other tissues and organs. In the study, 6 questions were asked on the relationship between systemic diseases and oral and dental health, and the scores were given correspondingly: the resident physicians averaged 90.3±14.4 points out of 100.

In the study conducted by Şenol et al^31^ on specialist medical doctors, the rate of correct answers to the field-specific questions examining the relationship between periodontal disease and systemic disease was found to be quite high (95.5% of internal medicine specialists, 80.6% of gynecologists and 86.2% of cardiologists answered the questions correctly). In the study conducted by Rabiei et al^25^ in Iran, the rate of correct answers to 9 questions posed to primary care physicians about the relationship between dentistry and systemic diseases was over 40%. In a study by Mohebbi et al,^19^ primary care physicians were asked questions about the relationship between diabetes mellitus and cardiovascular and periodontal disease; the questions were answered correctly at a high rate (63% to 97%).

Oral and dental health, as well as the health of other tissues and organs, is of great importance during pregnancy. In many studies, it has been shown that oral and dental health problems, especially periodontal disease and caries, are associated with some pregnancy complications in the prenatal period.^6,8,15^ Preventive, diagnostic and therapeutic dental care practices applied during pregnancy do not increase negative pregnancy outcomes.^29^ In fact, proper oral and dental care in the prenatal period can reduce pregnancy complications as well as reduce the risk of ECC (Early Childhood Caries). In this respect, it is important to evaluate all pregnant women in terms of oral and dental health in primary care family medicine practices, inform them about appropriate oral hygiene practices and refer them to the dentist when necessary.

The main methods determined to be positively related to the prevention of ECC are preventive dentistry visits and nutritional recommendations for children and pregnant women, prenatal oral health care, mother-and-child oral health programs implemented by family medicine specialists and dentists, and the use of varnishes, gels and toothpastes containing more than 1000 ppm fluoride for children.^4^ Considering the obvious benefits of protective F use and minimal side effects, it is recommended to start the use of fluoride toothpastes and F-containing varnishes with the eruption of the first teeth (6-8 months).^21,33^

Regarding the answers given to the mother-child-pregnancy (MCP) questions, the resident physicians averaged 61.1±15.8 points out of 100. There was a weak positive correlation between the ages of the resident physicians and their scores for the MCP questions (p<0.01 r = 0.256). MCP scores of married residents were statistically significantly higher than those of single residents (p<0.05). MCP scores of residents who had children were statistically significantly higher than those of residents who did not have children (p<0.05).

In the study conducted by Alshunaiber et al^3^ on family physicians and paediatricians in Saudi Arabia, the rate of correct answers to questions examining the relationship between MCP and oral and dental health was found to be 65.3%. In this study, similar to our study, the rate of giving correct answers to the question at which age should be started to use fluoride toothpaste in children was low (incorrect response rate 77.2%). In the study of Prakash et al^24^ on 1044 family physicians and paediatricians, physicians obtained 69 points out of 100 on their answers to the questions about ECC. In the study conducted by Forbes et al^11^ on family physician residents, the rate of correct answers to questions about pregnancy and ECC before education was 52.5%, while this rate increased to 75.7% after education. In the study conducted by Bayram et al,^5^ the ratio of family medicine residents’ correct answers to questions examining the relationship between MCP and oral and dental health was found to be higher in physicians who were married, had children, and worked longer in the profession, similar to our study. In the study conducted by Alshathri et al^2^ on family physicians in Saudi Arabia, the rate of correct answers to pediatric dentistry questions by female physicians was higher than male physicians.

In our study, the ratio of family medicine residents’ correct answers to questions about the relationship between MCP and oral and dental health was found to be moderate and low, similar to other studies. Unfortunately, a very low percentage of correct answers was given to the question asked about the application of F, which plays a crucial role in the prevention of ECC. This may be an indication that ECC and other child- and pregnancy-related oral and dental health problems are not sufficiently included in the medical curriculum and family medicine residency programs. In addition, physicians may not be up-to-date on information about oral and dental health during childhood and pregnancy.

Oral and dental health is one of the leading public health concerns worldwide. Four billion people (about half of the world’s population) are affected by oral and dental diseases. Oral and dental diseases affect the health level of society and individuals, in addition to posing a serious burden on the country’s economy. According to one analysis, in 2010, 300 billion dollars were spent globally only on dental diseases alone.^18^

As a requirement of the holistic approach, attention should be paid to oral and dental health while evaluating the general health status of individuals. In the study by Fotedar et al^12^ with primary health care providers in India, the average knowledge score was 51.9%. In the study conducted by Alshathri et al^2^ with family physicians in Saudi Arabia, the average knowledge score was found to be 68.5 out of 100. In the study conducted by Rabiei et al^25^ with primary care physicians, the mean knowledge score was found to be 53.5%.

According to the findings of our study, the general knowledge level of family medicine residents about oral and dental health was found to be moderate. Similar studies conducted in Turkey and abroad also support our findings. Family medicine practices, which constitute the most important part of primary health care practices in Turkey and in the world, can be seen as an opportunity to deliver oral and dental health services to all segments of society. First of all, the general health providers should have a high level of knowledge about oral and dental health. For this, oral and dental health education should be integrated into medical school curricula and medical specialisation (residency) education.

87.4% of the resident physicians participating in our study think that the mouth and teeth of the patients should also be checked during the oropharyngeal examination. In the study conducted by Sabbagh et al^27^ on 363 paediatricians, 78.8% of the physicians stated that they performed routine oral and dental health examinations. In the study of Alshunaiber et al^3^ on family physicians and paediatricians, 36.7% of family physicians and 63.3% of pediatricians think that children should be examined for caries.

In our study, 36.1% of the resident physicians were undecided about recommending the use of F, while 48.3% stated that they would recommend it. 57.7% of resident physicians stated that F application could be integrated into primary health care services. In the study by Di Guiseppe et al^9^ on paediatricians, 89% of physicians stated that they recommend the use of F to their patients. In the study by Gezgin et al^13^ on family physicians and dentists, 96% of the physicians stated that they recommend the use of topical F to their parents for their children. According to these studies, it is clear that especially paediatricians recommend the use of F for children more than do family physicians. Another finding of our study is that the knowledge of primary care physicians about the use of F is insufficient.

In our study, 78.2% of residents think that oral and dental health should be included in family medicine education and 81.5% in medical school education. At the same time, 80.8% of resident physicians stated that they did not receive oral and dental health education during family medicine residency programs. Although resident physicians did not receive oral and dental health education during medical school and residency education, they would have liked to.

## CONCLUSION

The general knowledge level of family medicine residents about oral and dental health was moderate in this study. Integrating oral and dental health issues into medical school, postgraduate and residency education programs is important and recommended in terms of contributing to the improvement of the oral- and dental-health care primary care physicians can provide.

## Figures and Tables

**Table:** 

	True	False	No idea
8) It is necessary to visit the dentist once a year.	☐	☐	☐
9) Teeth should be brushed 3 times a day.	☐	☐	☐
10) Toothbrush should be changed every 6 months.	☐	☐	☐
11) Dental floss should be used once a day.	☐	☐	☐
12) Gastroesophageal reflux disease can cause dental caries.	☐	☐	☐
13) One of the risk factors for gingivitis and periodontitis is diabetes mellitus.	☐	☐	☐
14) The patient diagnosed with diabetes mellitus should be directed to the dentist.	☐	☐	☐
15) The use of alcohol and cigarettes should be questioned in the patient with the complaint of non-healing wound in the oropharyngeal region.	☐	☐	☐
16) Drugs that cause dry mouth negatively affect oral and dental health. The patient should be informed about the use of these drugs.	☐	☐	☐
17) There is a direct relationship between respiratory diseases and oral health.	☐	☐	☐
18) Periodontal diseases can cause premature and low-weight baby delivery.	☐	☐	☐
19) Dental check-up is recommended for the patient who is planning a pregnancy.	☐	☐	☐
20) Dentist examinations are routinely required during pregnancy.	☐	☐	☐
21) Tooth or gum problems encountered during pregnancy should be postponed until after delivery.	☐	☐	☐
22) The incidence of gum disease increases during pregnancy.	☐	☐	☐
23) Oral and dental cleaning should be started after the first primary tooth erupts in the baby.	☐	☐	☐
24) The eruption of primary teeth is completed at the age of 6 years.	☐	☐	☐
25) The best food that can be given to a crying baby at night with a bottle is milk.	☐	☐	☐
26) Fluoride containing toothpastes can be used in children younger than 3 years old.	☐	☐	☐
27) In children, the first dental examination should be done if there is pain.	☐	☐	☐
28) Primary teeth do not need to be treated.	☐	☐	☐
29) Untreated cavities in children can affect overall health.	☐	☐	☐
30) Toothbrushing training should be given after all teeth have erupted.	☐	☐	☐
31) The bacteria that cause dental caries most are Streptococcus mutans and Lactobacilli.	☐	☐	☐
32) Bacteria that cause tooth decay can be passed from mother to baby.	☐	☐	☐
33) A child whose tooth is broken as a result of trauma should be directed to the dentist after placing the broken tooth piece in sterile water or milk.	☐	☐	☐

**Table:** 

	Strongly disagree	Disagree	Undecided	Agree	Strongly Agree
34) The family medicine specialist should also check the patient’s mouth and teeth during the oropharyngeal examination of the patients.	☐	☐	☐	☐	☐
35) I recommend the use and/or application of fluoride to the patient who wants information about the use and application of fluoride.	☐	☐	☐	☐	☐
36) Oral and dental care (brushing, flossing, etc.) should be explained by assistant health personnel in family health centers.	☐	☐	☐	☐	☐
37) Oral and dental screening and fluoride application should be integrated into primary health care services.	☐	☐	☐	☐	☐
38) Oral and dental health should be integrated into the family medicine specialty curriculum.	☐	☐	☐	☐	☐
39) Oral and dental health should be given more place in medical school education.	☐	☐	☐	☐	☐
40) During my family medicine residency training, I received oral and dental health training.	☐	☐	☐	☐	☐
41) Oral and dental health is adequately covered in the basic books of family medicine. Tablet Varnish Addition to drinking water Gargle Toothpaste	☐	☐	☐	☐	☐

## Supplementary material (Questionnaire)

If your answer to Question 35 is “agree” or “strongly agree,” in what form(s) would you recommend the use of fluoride? (you may select more than one option)

1) Age?

____________

2) Gender?

1. Female ☐
2. Male ☐

3) Marital status?

1. Married ☐
2. Single ☐
3. Divorced ☐

4) Do you have children?

1. Yes ☐
2. No ☐

5) How many years in professional life?

1. 0-1 years ☐
2. 2-3 years ☐
3. 4-5 years ☐
4. 6-10 years ☐
5. >10 years ☐

6) Institution?

1. Training and research hospital ☐
2. Faculty of medicine ☐

7) Have you ever worked in a family health center before?

1. Yes ☐
2. No ☐
